# Effects of nusinersen on motor function in children with spinal muscular atrophy: a retrospective study

**DOI:** 10.3389/fneur.2024.1391613

**Published:** 2024-07-15

**Authors:** Yuyi Chen, Dongling Yang, Xuelin Huang, Juntan Feng, Qingqing Zhao, Huixian Huang, Lushi Liang, Xinxin Zhang, Yiyan Ruan

**Affiliations:** Department of Pediatric Neurology, Maternal and Child Health Hospital of Guangxi Zhuang Autonomous Region, Nanning, China

**Keywords:** spinal muscular atrophy, nusinersen, children, motor function, effects

## Abstract

**Background:**

Spinal muscular atrophy (SMA) is a genetic progressive neuromuscular disease. Nusinersen is the first disease modifying drug approved to treat patients with SMA. Our study aimed to evaluate the efficacy of nusinersen treatment on motor function in children with SMA.

**Methods:**

A retrospective analysis was conducted on the data of 52 genetically confirmed SMA patients from November 2020 to September 2023. Motor function was assessed based on standardized scales from baseline to 14 months of follow-up.

**Results:**

Of patients in this study, the majority had SMA type 2 (40/52, 76.9%), 5 (9.6%) and 7 (13.5%) patients had SMA types 1 and 3, respectively. The median disease duration was 11 months (range 0–52), and the median age at initiation of treatment was 44.5 months (range 5–192). Motor function of all the patients with SMA improved from baseline to 14 months of follow-up. Mean increases of 4.6-point (*p* = 0.173), 4.7-point (*p* = 0.021) and 2.7-point (*p* = 0.013) were observed from baseline to 14 months of follow-up for the Children’s Hospital of Philadelphia Infant Test of Neuromuscular Disorders scores, the Hammersmith Functional Motor Scale Expanded (HFMSE) and the Revised Upper Limb Module (RULM), respectively. Increased disease duration and age of treatment initiation were negatively correlated with the changes in HFMSE scores (*r* = −0.567, *p* = 0.043; *r* = −0.771 and *p* = 0.002, respectively). Similar results were observed for the RULM scores (*r* = −0.714, *p* = 0.014; *r* = −0.638 and *p* = 0.035, respectively).

**Conclusion:**

Our study suggested that 14 months of treatment with nusinersen was effective and improved the motor function of children with SMA types 1, 2, or 3. In addition, disease duration and age at treatment initiation were negatively correlated with treatment outcome in the patients.

## Introduction

Spinal muscular atrophy (SMA) is a rare inherited neuromuscular disorder characterized by progressive muscle atrophy, weakness and paralysis (1). Approximately 1 in 100,000 people worldwide suffer from SMA, with an incidence of 1 in 10,000 live births (2). SMA is caused by insufficient survival motor neuron (SMN) protein levels due to either a homozygous deletion or mutation in the *SMN1* gene, which results in the degeneration of motor neurons in the anterior horn cells of the spinal cord (1, 3). SMA is clinically classified into five types (0–4) based on age at onset and maximal motor function achieved (4, 5). The *SMN2* gene is a major SMA disease-modifying gene with high homology to the *SMN1* gene (1, 3). Most transcripts of the SMN2 gene lack the exon 7 encoding and is truncated resulting in non-functioning SMN proteins, with only 10% of the full-length proteins being synthesized (1, 3). It is known that SMN2 copy numbers are correlated with disease severity (6).

In recent years, several drugs have been developed for SMA and these primarily target SMN1 and SMN2. Nusinersen is the first SMA disease-modifying medication that was approved by the United States Food and Drug Administration and the European Medicines Agency in 2016 and 2017, respectively. Nusinersen received its Chinese Drug Administration approval for the treatment of SMA in 2019. It is an antisense oligonucleotide (ASO) intrathecal drug that increases SMN2 exon 7 inclusion and regulates SMN2 pre-mRNA splicing in order to produce a functional SMN protein (7). Previous studies have demonstrated that nusinersen effectively improved motor function in both pediatric and adult SMA patients (8, 9).

A progressive disease course in all types of SMA has been shown in natural history studies. Nusinersen has the advantage of being fast-acting in the treatment of SMA. However, real-world data on the effectiveness of nusinersen treatment is limited, especially with relatively few studies on the correlation between improved motor function and disease duration as well as age at treatment initiation of the disease. This retrospective study aimed to investigate the efficacy of nusinersen in children with SMA with different treatment durations for up to 14 months.

## Patients and methods

### Participants

This retrospective study included all pediatric patients with SMA treated with intrathecal nusinersen in the Department of Pediatric Neurology, Maternal and Child Health Hospital of Guangxi Zhuang Autonomous Region from November 2020 to September 2023. The study was approved by the Ethics Committee of our hospital in accordance with the Declaration of Helsinki. Informed consent was obtained from the parents or guardians of the patients involved in this study.

The inclusion criteria were as follows: (1) genetically confirmed diagnosis of SMA (homozygous deletion or compound heterozygous mutation of SMN1 gene); (2) clinically confirmed diagnosis of SMA; (3) age <18 years, and (4) the minimum and maximum treatment duration is between 2 [M2] and 14 [M14] months, respectively. The exclusion criteria were as follows: (1) lack of definitive genetic testing; (2) diagnosis of other neurological/neuromuscular diseases; (3) history of treatment with other medications for SMA, and (4) incomplete clinical information.

Intrathecal injections of nusinersen were given from the loading phase (at days 0, 14, 28, and 63) and followed by injections every 4 months thereafter, according to the treatment protocol. Sociodemographic and clinical data recorded at baseline for the SMA patients included sex, age, SMA type, SMN2 gene copy number, motor status, family history of SMA, presence of dysarthria/hyperreflexia, presence of scoliosis, presence of arthrogryposis and use of ventilation. Follow-up data were collected at months 2 [M2], 6 [M6], 10 [M10], and 14 [M14] of nusinersen treatment.

### Functional assessment

Motor function at baseline and post-treatment was assessed by a professional physiotherapist using different scales depending on the patient’s age and SMA type. The World Health Organization (WHO) motor milestones were used to assess gross motor development in patients with SMA types 1 and 2 (10). The Children’s Hospital of Philadelphia Infant Test of Neuromuscular Disorders (CHOP-INTEND) was available for all patients younger than 2 years and all non-sitters (11). The sitters at 2 years of age and older underwent assessment by the Hammersmith Functional Motor Scales Expanded (HFMSE) (12). The Revised Upper Limb Module (RULM) was applied to measure motor function of the upper extremities in most SMA patients (13). Motor function from included SMA patients was assessed at each time point of nusinersen treatment and these were compared to the baseline values.

### Statistical analysis

The statistical analysis was performed by SPSS software version 26 and Microsoft Excel 2016. Categorical variables were expressed as frequencies and percentages. Data was tested for normality using the Shapiro–Wilk test. Normally distributed data were presented as means ±SDs, whereas data with non-normal distribution were presented as medians [interquartile ranges (IQR)]. Changes in motor function were analyzed after 2, 6, 10, and 14 months of nusinersen treatment (designated M2, M6, M10, and M14, respectively) with the Wilcoxon signed rank test. Pearson’s correlation coefficient was used to assess the correlations between changes in motor function from baseline to M14 and either disease duration or age at treatment initiation. A statistically significant difference was defined as *p* < 0.05.

## Results

### Patients’ characteristics

Fifty-three patients with genetically confirmed SMA were treated with nusinersen from November 2020 to September 2023 in our hospital. Among them, 52 patients were included in the study, of which 1 was excluded due to a history of treatment with risdiplam. The baseline characteristics of included patients are presented in Table 1. Among the 52 patients, 20 (38.5%) were females and 32 (61.5%) were males. The median ages of the patients at symptom onset, disease duration and at treatment initiation were 8 months (range 1–120), 11 months (range 0–52), and 44.5 months (range 5–192), respectively. Most of patients had SMA type 2 (40/52, 76.9%) and 5 (9.6%) had SMA type 1, and 7 patients (13.5%) had SMA type 3. Besides, 5 (9.4%) patients had a family history of SMA. Genetic testing showed that all patients had a homozygous deletion of *SMN1*. 4 (7.7%) patients had 2 copies of *SMN2*, 46 (88.5%) patients had 3 copies of *SMN2* and *2* (3.8%) patients had 4 copies of *SMN2*. Of 52 patients were assessed for motor milestones at baseline, 30 (57.7%) could sit without support, 8 (15.4%) stood alone and 4 (7.7%) walked alone (Table 1). Areflexia/hyporeflexia was manifested in all the patients. 37 (71.2%) patients had scoliosis, 12 (23.1%) had arthrogryposis, and 8 (15.4%) required ventilation.

**Table 1:** Baseline demographic and clinical characteristics of the SMA patients in this study.

Parameter	SMA type 1 *n* = 5 (9.6%)	SMA type 2 *n* = 40 (76.9%)	SMA type 3 *n* = 7 (13.5%)	Total *n* = 52 (100%)
Sex, *n* (%)				
Female	1 (20)	17 (42.5)	2 (28.6)	20 (38.5)
Male	4 (80)	23 (57.5)	5 (71.4)	32 (61.5)
Age (month, median, range)				
Age at symptom onset	3 (1, 5)	8 (3, 19)	24 (12, 120)	8 (1, 120)
Age at diagnosis	9 (4, 12)	19 (4, 64)	30 (19, 160)	19.5 (4, 160)
Age at treatment initiation	10 (5, 18)	45 (8, 192)	107 (40, 162)	44 (5, 192)
Disease duration	4 (3, 8)	9 (0, 52)	8 (4, 40)	8 (0, 52)
*SMN2* copy number, *n* (%)				
2	3 (60)	1 (2.5)	0	4 (7.7)
3	2 (40)	39 (97.5)	5 (71.4)	46 (88.5)
4	0	0	2 (28.6)	2 (3.8)
Family history of SMA, *n* (%)	0	5 (12.1)	0	5 (9.4)
Motor status, *n* (%)				
Sitting without support	0	23 (57.5)	7 (100)	30 (57.7)
Stand alone	0	1 (2.5)	7 (100)	8 (15.4)
Walk alone	0	0	4 (57.1)	4 (7.7)
Areflexia/hyporeflexia, *n* (%)	5 (100)	40 (100)	7 (100)	52 (100)
Scoliosis, *n* (%)	2 (40)	30 (75)	5 (71.4)	37(71.2)
Arthrogryposis, *n* (%)	1 (20)	11 (27.5)	0	12 (23.1)
Ventilation, *n* (%)	3 (60)	5 (12.5)	0	8 (15.4)

Data are presented as either medians (ranges) or *n* (%), unless otherwise specified. Disease duration = age at diagnosis minus age at symptom onset. SMA, spinal muscular atrophy; n, number; SMN2, survival of motor neuron 2 gene.

### Motor function

**Figure 1:**
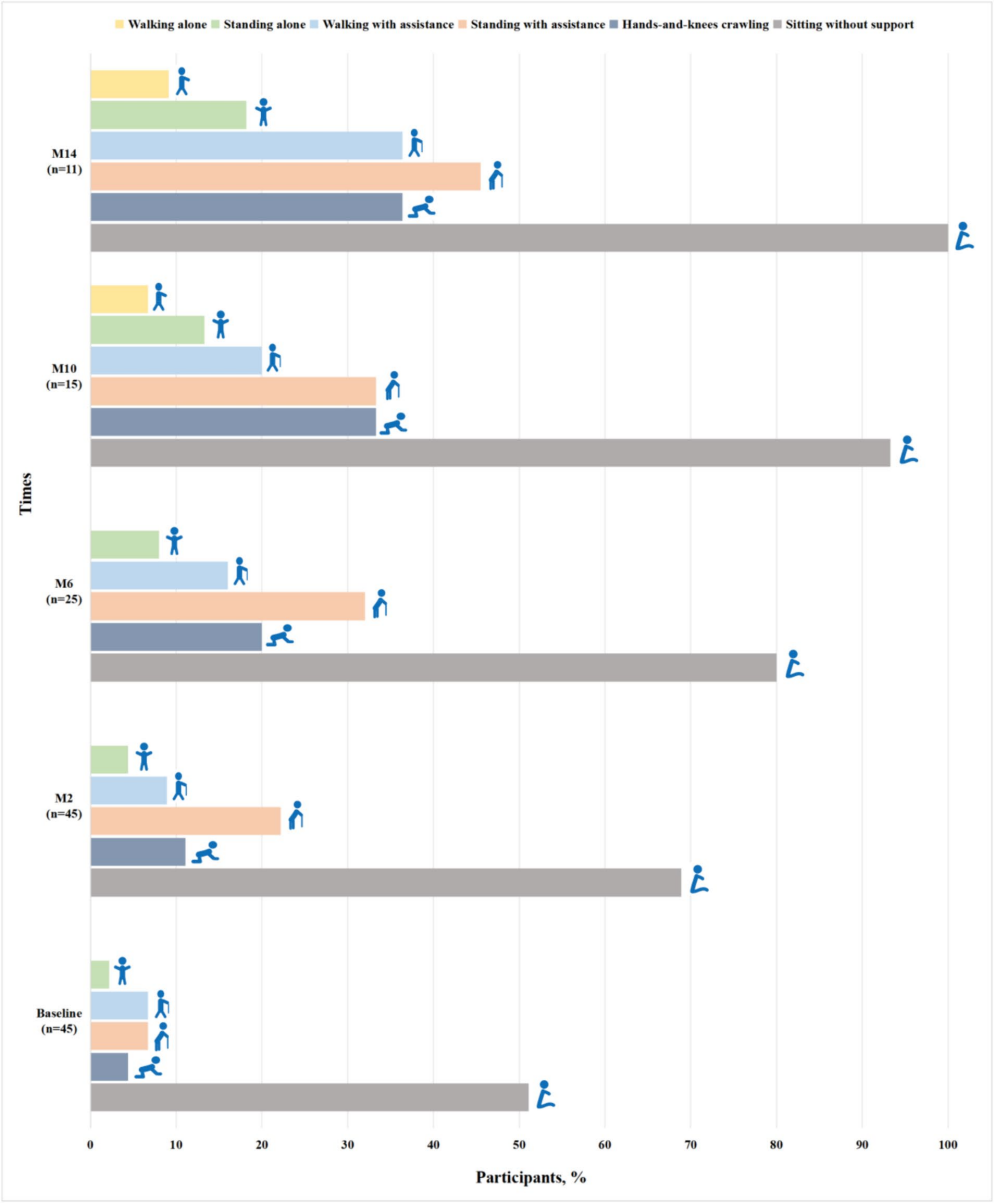
Proportions of participants with SMA types 1 and 2 achieving WHO motor milestones when assessed by the duration of nusinersen treatment (M2, M6, M10, and M14 refer to 2, 6, 10, and 14 months of treatment, respectively).

Among 52 patients assessed for WHO motor milestones at baseline, 7 patients with SMA type 3 achieved all motor milestones. Of 45 patients with SMA types 1 and 2, 23 (51.1%) could sit without support, 2 (4.4%) achieved hands-and-knees crawling, 3 (6.7%) stood with assistance, 3 (6.7%) walked with assistance and 1 (2.2%) stood alone (Figure 1). Gross motor function gradually improved at subsequent time points of nusinersen treatment. Up to M14, all the patients (11/11) could sit without support, 36.4% (4/11) achieved hands-and-knees crawling, 45.5% (5/11) stood with assistance, 36.4% (4/11) walked with assistance, 18.2% (2/11) stood alone and one patient achieved all motor milestones (Figure 1).

**Figure 2:**
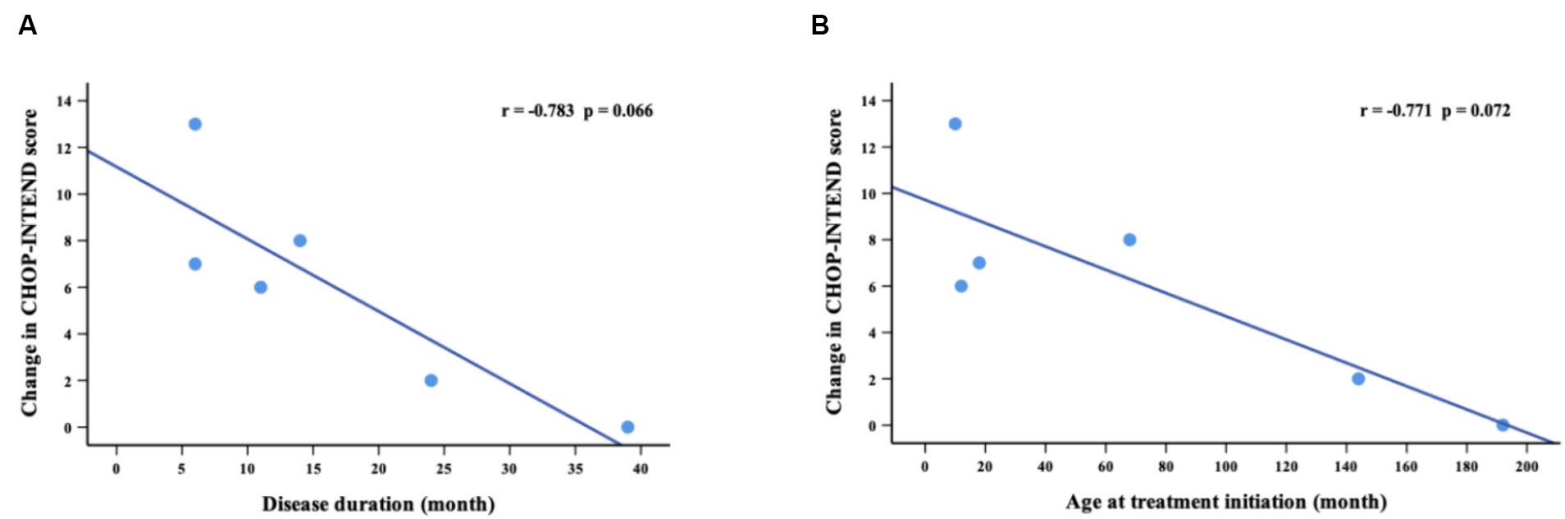
Correlations between change in CHOP-INTEND scores (from baseline to M14) and disease duration **(A)** and age at treatment initiation **(B)**. M14 represents 14 months of nusinersen treatment.

The CHOP-INTEND assessment at baseline was performed in 14 non-sitters, including 3 with SMA type 1 and 11 with SMA type 2. During the nusinersen treatment, the percentage of patients who improved increased to 36.4% (4/11) at M2, 28.6% (2/7) at M6, 66.7% (4/6) at M10 and 83.3% (5/6) at M14 (Table 2). A clinically meaningful improvement in CHOP-INTEND scores (≥4 points) was observed in 27.3% of patients at M2, 28.6% at M6, 33.3% at M10 and 66.7% at M14 (Supplementary Table S1). The mean CHOP-INTEND score at baseline was 30.9 points, which increased at each time points of nusinersen treatment and reached 33.7 points at M14. The mean value of the change in CHOP-INTEND scores between baseline and 2 months was 0.2 points, which increased to 4.6 points by 14 months. However, no significant differences in CHOP-INTEND scores were found between baseline and each time point of nusinersen treatment. There was also no correlation between the change in CHOP-INTEND score and disease duration or age at treatment initiation (*r* = −0.783, *p* = 0.066 and *r* = −0.771, *p* = 0.072, respectively) (Figure 2).

**Table 2:** Changes in CHOP-INTEND scores when assessed by the duration of nusinersen treatment in SMA patients (M2, M6, M10, and M14 refer to 2, 6, 10, and 14 months of treatment, respectively).

	*N*	Mean CHOP-INTEND score	Improved (%)	Mean change from baseline	*p*-value
Baseline	14	30.9 ± 14.5 (3, 60)	NA	NA	NA
M2	11	33.4 ± 15.3 (3, 60)	4 (36.4)	0.2 ± 3.9 (−8,6)	0.230
M6	7	33.7 ± 17.3 (3, 60)	2 (28.6)	0.7 ± 4.2 (−7,5)	0.176
M10	6	31.0 ± 19.8 (3, 62)	4 (66.7)	1.8 ± 1.8 (0,4)	0.207
M14	6	33.7 ± 19.6 (3, 62)	5 (83.3)	4.6 ± 3.4 (0,4)	0.173

*N*, number of SMA patients included; CHOP-INTEND, Children’s Hospital of Philadelphia Infant Test of Neuromuscular Disorder; NA, not applicable.

**Figure 3:**
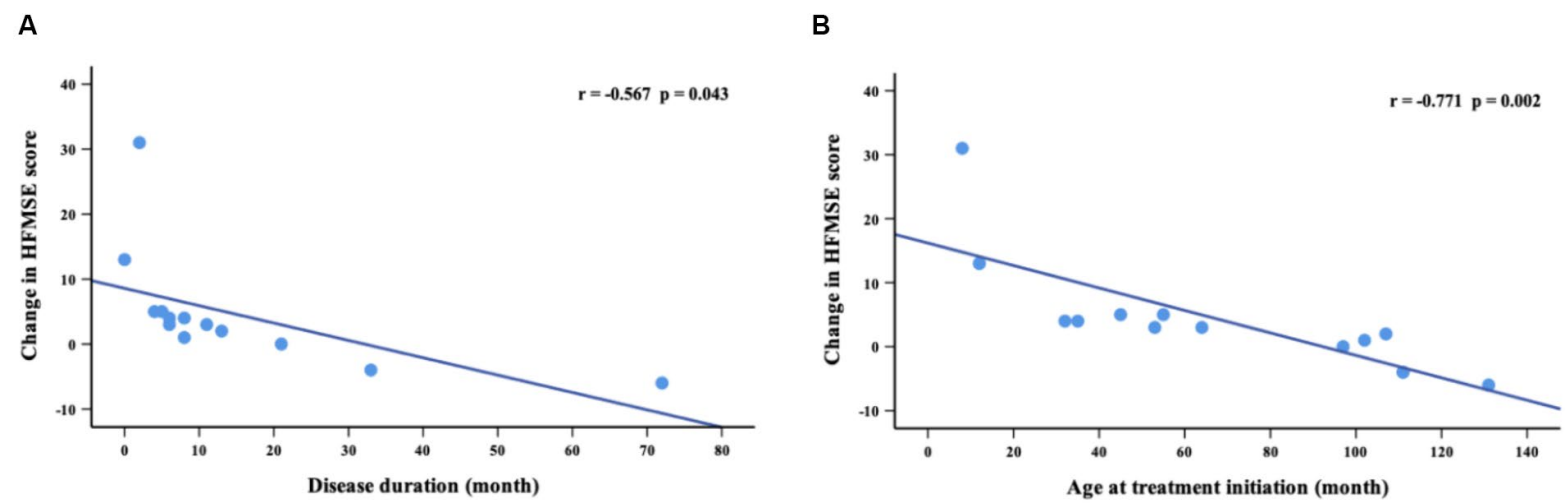
Correlation between change in HFMSE scores (from baseline to M14) and disease duration **(A)** and age at treatment initiation **(B)**.

Of 39 patients assessed with HFMSE at baseline, 32 patients had SMA type 2 and 7 had SMA type 3. Compared with baseline, 59. 1% of patients (13/22) at M2, 50.0% (8/16) at M6, 69.2% (9/13) at M10 and 76.9% (10/13) at M14 showed improvements in their condition (Table 3). 27.3% of patients (6/22) at M2, 43.8% (7/15) at M6, 53.8% (7/11) at M10 and 61.5% (8/11) at M14 showed clinically significant improvements in HFMSE scores (≥3 points) (Supplementary Table S2). The mean HFMSE score at baseline was 23.4 points, which increased incrementally at subsequent time points of nusinersen treatment and reached 28.2 points at 14 months. The mean value of the change in HFMSE scores between baseline and 2 months was 1.3 points, which increased to 4.7 points by 14 months. There were significant differences in HFMSE scores from baseline to M2, M6, M10, and M14 (*p* = 0.001, *p* = 0.002, *p* = 0.019 and *p* = 0.021, respectively). The change in HFMSE scores from baseline to M14 showed a negative correlation with disease duration and age at treatment initiation (*r* = −0.567, *p* = 0.043 and *r* = −0.771, *p* = 0.002, respectively) (Figure 3).

**Table 3:** Changes in HFMSE scores when assessed by the duration of nusinersen treatment in SMA patients (M2, M6, M10, and M14 refer to 2, 6, 10, and 14 months of treatment, respectively).

	*N*	Mean HFMSE score	Improved (%)	Mean change from baseline	*p*-value
Baseline	39	23.4 ± 13.0 (0, 59)	N/A	N/A	N/A
M2	22	30.3 ± 14.0 (0, 63)	13 (59.1)	1.3 ± 3.8 (−9,11)	0.001
M6	16	30.0 ± 16.3 (4, 64)	8 (50.0)	2.4 ± 7.2 (−8,19)	0.002
M10	13	28.3 ± 15.5 (4, 60)	9 (69.2)	4.0 ± 7.7 (−1,26)	0.019
M14	13	28.2 ± 13.7 (5, 54)	10 (76.9)	4.7 ± 9.1 (−6,31)	0.021

*N*, number of SMA patients included; HFMSE, Hammersmith Functional Motor Scales Expanded; NA, not applicable.

**Figure 4:**
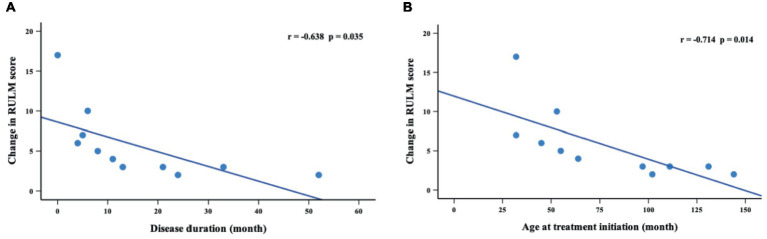
Correlations between change in RULM scores (from baseline to M14) and disease duration **(A)** and age at treatment initiation **(B)**. M14 represents 14 months of nusinersen treatment.

Thirty-six patients underwent RULM assessment at baseline, of which 29 patients had SMA type 2 and 7 patients had SMA type 3. During the nusinersen treatment, improvement was found in 50.0% of patients (11/22) at M2, 60.0% (9/15) at M6, 81.8% (9/11) at M10 and 90.9% (10/11) at M14 (Table 4). The percentage of patients who achieved clinically meaningful improvements (≥2 points) in RULM was 40.9% (9/22) at M2, 53.3% (8/15) at M6, 81.8% (9/11) at M10 and 90.9% (10/11) at M14 (Supplementary Table S3). The mean RULM score at baseline was 19.3 points, which increased at each time point of nusinersen treatment and reached 24.4 points at M14. The mean value of the change in RULM scores between baseline and 2 months was 1.6 points, which increased to 2.7 points at 14 months. Significant differences in RULM scores were observed from baseline to M2, M6, M10, and M14 (*p* = 0.004, *p* = 0.027, *p* = 0.033 and *p* = 0.013, respectively). The change in RULM score was negatively correlated with the increase in disease duration and treatment initiation (*r* = −0.714, *p* = 0.014 and *r* = −0.638, *p* = 0.035, respectively) (Figure 4).

**Table 4:** Changes in RULM scores when assessed by the duration of nusinersen treatment in SMA patients (M2, M6, M10, and M14 refer to 2, 6, 10, and 14 months of treatment, respectively).

	*N*	Mean RULM score	Improved (%)	Mean change from baseline	*p*-value
Baseline	36	19.3 ± 10.3 (4, 37)	N/A	N/A	N/A
M2	22	23.0 ± 12.3 (5, 37)	11 (50.0)	1.6 ± 4.3 (−4,13)	0.004
M6	15	21.0 ± 12.2 (0, 37)	9 (60.0)	1.4 ± 5.5 (−8,15)	0.027
M10	11	23.4 ± 9.3 (9, 37)	9 (81.8)	1.7 ± 4.7 (−7,7)	0.033
M14	11	24.4 ± 9.8 (9, 37)	10 (90.9)	2.7 ± 5.1 (−9,10)	0.013

*N*, number of SMA patients included; RULM, Revised Upper Limb Module; NA, not applicable.

## Discussion

The SMA is a progressive degenerative neuromuscular disease, leading to muscle weakness and even respiratory insufficiency in the most severe cases (1). Nusinersen is a fast-acting ASO drug that provides benefit to patients during the early stages of their treatment for this condition (7). Our study demonstrated the therapeutic effect of nusinersen on motor function in children with SMA types 1, 2, and 3. Despite a wide age range, varying functional capacity at treatment initiation and different comorbidities, in the majority of children their disease progression was stabilized and they benefited from nusinersen treatment.

The natural history data of SMA type 1 patients without drug treatment showed a gradual decline in CHOP-INTEND scores irrespective of their age and baseline scores (14). In addition, Coratti et al. (15, 16) revealed that 3.1% of SMA type 2 patients lost the ability to sit independently within 12 months, and a decline in both HFMSE and RULM scores in SMA types 2 and 3 patients was observed. Therefore, stabilization of disease progression should be seen as a positive response to treatment. The ENDEAR clinical trial showed 71% responders (improvement of ≥4 points in CHOP-INTEND scores) in patients with SMA type 1 after 13 months of nusinersen treatment (8). Pechmann et al. (17) observed a significant change in CHOP-INTEND scores from baseline values. In our study, a response rate of 36.4% was observed at 2 months and this increased to 83. 3% at 14 months. The mean CHOP-INTEND scores increased from baseline at each time point of nusinersen treatment, which was similar to results observed in previous studies. No significant differences were found in the CHOP-INTEND scores between baseline and each time point of nusinersen treatment. In addition, there was no statistically significant correlation between improvement in CHOP-INTEND scores and either disease duration or age at treatment initiation. This is most likely due to the small number of patients included in this study.

The CHERISH study found that HFMSE scores improved by an average of 4 points after 15 months of nusinersen treatment (18). Szabó et al. (19) found a 7-point improvement in HFMSE scores at the day 429 visit. Our data presented an increase of 4.7 points in HFMSE scores after 14 months of follow-up. Additionally, the difference in mean HFMSE scores was significant from baseline to 14 months. We also found that the change in HFMSE scores was significantly negatively correlated with disease duration and age at treatment initiation in children with SMA types 2 and 3. Another prospective 3-year study reflected that RULM scores improved in all pediatric patients with SMA types 2 and 3 (20). Mercuri et al. (18) found a mean increase of 1.31 points as assessed by RULM scores after 2 months of treatment in patients unable to walk. Similarly, we observed an increase in RULM scores of 1.6 at 2 months and 4.7 at 14 months in children with SMA types 2 and 3. There were significant differences in RULM scores from baseline at each time point of nusinersen treatment. Patients showed increases in RULM scores with increased disease duration and age at treatment initiation.

For SMA patients able to walk unsupported at least 10 meters, the six-minute walk test (6MWT) was used to assess activity endurance (21). In our study, one patient with SMA type 3 performed the 6MWT. A 7.5-meter increase in 6WMT was found from 10 to 14 months, which is consistent with a study that patients with SMA type 3 had a median increase in walking distance of 17 m at day 253 (22). Szabó et al. (19) also observed improvements in patients undergoing the 6WMT after 63 days of nusinersen treatment. In contrast, Croatian real-world data showed no significant improvement in 6WMT in SMA type 3 patients who were introduced to nusinersen at >18 years of age (23). More research is still needed on the efficacy of nusinersen treatment in paediatric and adults with SMA type 3. In addition, we observed that SMA types 1 and 2 patients had marked improvement in the WHO motor milestones from baseline to 14 months, especially for sitting without support and standing with assistance. More importantly, none of the patients showed regression in the WHO motor milestones. Another study in SMA types 1 and 2 patients also found slight improvement in the WHO milestone in terms of sitting alone and crawling with hands and knees after 2 months of nusinersen treatment (24). However, relatively fewer studies have been conducted using the WHO motor milestones in pediatric patients with SMA following nusinersen treatment.

Taken together, our data suggested that SMA children showed an overall improvement in motor function after nusinersen treatment. Due to the limited number of patients, our study did not evaluate motor function based on different types of SMA, making it difficult to better describe the effects of nusinersen and treatment differences among the patients. It is known that the copy number of the *SMN2* gene may be an influencing factor in treatment efficacy (25, 26). Likewise, we were unable to explore the effect of *SMN2* gene copy number owing to a small patient population. In addition, the modified Hammersmith infant neurologic examination-part 2 (HINE-2) was not included in the clinical assessment followed in our study and this test could better assess motor development in SMA type 1 patients with different subtypes (27, 28). Therefore, it is necessary to include more patients for longer follow-up studies in future.

## Conclusion

In brief, our study found that nusinersen treatment was effective in children with SMA types 1, 2, and 3. Improvement in motor function in SMA patients was observed after 14 months of nusinersen treatment. Moreover, disease duration and age at treatment initiation were negatively correlated with treatment outcome. Our findings will contribute to the clinical management and efficacy assessment of nusinersen therapy in future patients suffering from SMA. In addition, further research is needed to analyze the long-term effects of nusinersen treatment and improve the survival and life quality of patients with SMA.

## Data availability statement

The raw data supporting the conclusions of this article will be made available by the authors, without undue reservation.

## Ethics statement

The studies involving humans were approved by the Ethics Committee of Maternal and Child Health Hospital of Guangxi Zhuang Autonomous Region. The studies were conducted in accordance with the local legislation and institutional requirements. Written informed consent for participation was not required from the participants or the participants’ legal guardians/next of kin in accordance with the national legislation and institutional requirements.

## Author contributions

YC: Conceptualization, Data curation, Formal analysis, Writing – original draft. DY: Conceptualization, Data curation, Formal analysis, Writing – original draft. XH: Data curation, Investigation, Writing – original draft. JF: Data curation, Investigation, Writing – original draft. QZ: Data curation, Writing – original draft. HH: Data curation, Writing – original draft. LL: Data curation, Writing – original draft. XZ: Data curation, Writing – original draft. YR: Conceptualization, Data curation, Funding acquisition, Investigation, Writing – review & editing.
