# Supplementary material for: Effects of nusinersen on motor function in children with spinal muscular atrophy: a retrospective study
Source: Front Neurol. 2024 Jul 15;15:1391613. doi: 10.3389/fneur.2024.1391613 (PMC11284043; doi:10.3389/fneur.2024.1391613)
Supplement: Supplementary file 1 [file Table_1.DOCX]

**Supplementary materials**

| **Supplementary Table 1** Proportions of participants achieving CHOP-INTEND improvement scores ≥ 4 when assessed by the duration of nusinersen treatment (M2, M6, M10 and M14 refer to 2, 6, 10 and 14 months of treatment, respectively). | | |
| --- | --- | --- |
|  | **N** | **Improved ≥ 4 points (%)** |
| Baseline | 14 | NA  3 (27.3)  2 (28.6)  2 (33.3)  4 (66.7) |
| M2 | 11 |  |
| M6 | 7 |  |
| M10 | 6 |  |
| M14 | 6 |  |
| *N* number of SMA patients included, *CHOP-INTEND* Children’s Hospital of Philadelphia Infant Test of Neuromuscular Disorder, *NA* not applicable | | |

| **Supplementary Table 2** Proportions of participants achieving HFMSE improvement scores ≥ 3 when assessed by the duration of nusinersen treatment (M2, M6, M10 and M14 refer to 2, 6, 10 and 14 months of treatment, respectively). | | |
| --- | --- | --- |
|  | **N** | **Improved ≥ 3 points (%)** |
| Baseline | 39 | NA  6 (27.2)  7 (43.8)  7 (53.8)  8 (61.5) |
| M2 | 22 |  |
| M6 | 15 |  |
| M10 | 13 |  |
| M14 | 13 |  |
| *N* number of SMA patients included, *HFMSE* Hammersmith Functional Motor Scales Expanded, *NA* not applicable | | |

| **Supplementary Table 3** Proportions of participants achieving RULM improvement scores ≥ 2 when assessed by the duration of nusinersen treatment (M2, M6, M10 and M14 refer to 2, 6, 10 and 14 months of treatment, respectively). | | |
| --- | --- | --- |
|  | **N** | **Improved ≥ 2 points (%)** |
| Baseline | 36 | NA  11 (50.0)  9 (60.0)  9 (81.8)  10 (90.9) |
| M2 | 22 |  |
| M6 | 15 |  |
| M10 | 11 |  |
| M14 | 11 |  |
| *N* number of SMA patients included, *RULM* Revised Upper Limb Module, *NA* not applicable | | |
